# A pilot randomized trial on the usability and acceptability of an app (MyIBDDiet) to improve the self-management of anti-inflammatory diet for individuals with inflammatory bowel disease: A protocol paper

**DOI:** 10.1371/journal.pone.0353123

**Published:** 2026-07-02

**Authors:** Ravneet Kaur, Kendall van Diepen, Somayeh Raiesdana, Kaitlyn Delaney Chappell, Lekan Ajibulu, Michal Gozdzik, Brendan Halloran, Frank Hoentjen, Karen I. Kroeker, Farhad Peerani, Carla M. Prado, Dina Kao, Karen Wong

**Affiliations:** 1 Department of Medicine, Division of Gastroenterology, Faculty of Medicine and Dentistry, University of Alberta, Alberta, Canada; 2 Department of Food and Nutrition Science, Faculty of Agricultural, Life and Environmental Science, University of Alberta, Alberta, Canada; Instituto Nacional de Ciencias Medicas y Nutricion Salvador Zubiran, MEXICO

## Abstract

The role of diet in the management of inflammatory bowel disease (IBD) is increasingly recognized with recent guidelines providing specific dietary recommendations. Although mobile health apps targeting diet and lifestyle habits in IBD are emerging, few are designed for self-management or have been formally evaluated for effectiveness. We have co-designed a diet guidance and tracking app (MyIBDDiet) with and for patients with IBD with the aim of improving overall diet profile. We will be conducting a 60-day single-centre pilot randomized trial of 40 IBD patients randomized in 1:1 ratio to MyIBDDiet app or usual care. Participants in the usual care group will crossover to the MyIBDDiet app group after 30 days. Primary outcome is usability assessed using a mixed method quantitative [Theoretical Framework of Acceptability (TFA), mHealth App Usability Questionnaire (MAUQ)], and qualitative approach (semi-structured interviews). Secondary outcomes include clinical efficacy evaluated by change in diet quality [Mini-EAT questionnaire, Automated Self-administered 24-Hour Dietary Assessment Tool (ASA-24), Healthy Eating Index (HEI), Mediterranean Diet Serving Score (MDSS)], changes in biomarkers of processed food intake (spot urine sodium and chloride), changes in IBD disease activity [Patient Reported Outcome (PRO2 and PRO3), C-reactive protein, fecal calprotectin], changes in quality of life [EuroQol-5 Dimension (EQ-5D), Short Inflammatory Bowel Disease Questionnaire (SIBDQ)] and safety. Exploratory outcomes include changes in fecal microbiome and serum and fecal metabolome. Additional quantitative data will be collected from the digital analytics of MyIBDDiet app. The pilot data generated will inform the design of an adequately powered randomized trial and future mobile app development and evaluation by providing a framework for evaluation of clinical effectiveness.

**Trial registration:**

ClinicalTrials.gov Identifier: NCT06683105. Registered on 8 November 2024.

## Introduction

Inflammatory bowel disease (IBD) comprising of Crohn’s disease and ulcerative colitis, is characterized by chronic inflammation of the gastrointestinal tract due to an abnormal immune response to gut microbiota in genetically susceptible individuals [1]. Canada has among the highest burden of IBD in the world with a forecasted prevalence reaching 1.1% (470,000) by 2035 [2].

Food and nutrition have a profound impact on the lives of people living with IBD due to the impact of eating on symptoms, the psychosocial impact of food avoidance, the high prevalence of nutrition‐related problems and the role of nutrition in the management of disease [3–5]. One study demonstrated that the majority of participants (85.4%) believed that diet could trigger IBD relapses [6]. According to Bergeron et al. 2018, food exclusion rates are 69% higher in people experiencing a flare versus those in remission [7]. Food avoidance and restrictive diet patterns have been correlated with higher risk of malnutrition and poorer food related quality of life [8–10]. The intestinal involvement and inflammation further contribute to the high prevalence of malnutrition ranging between 20 and 85% in IBD patients with protein energy malnutrition and weight loss being the most common during active disease [11–13].

Nutritional therapies in IBD include therapeutic diet strategies to induce remission of active IBD and general recommendations to improve overall health and well-being [14]. Prospective cross-sectional cohort studies have linked the consumption of ultra-processed foods to the onset of IBD and among patients with diagnosis of IBD, a higher risk of IBD-related surgeries [15–17]. The evidence for induction of remission is strongest for exclusive enteral nutrition (EEN), particularly in the pediatric population, and the Crohn’s disease exclusion diet (CDED) [18,19]. However, these dietary treatments are challenging to follow and require guidance of a dietitian. There is emerging evidence to support anti-inflammatory diets such as Mediterranean Diet and Specific Carbohydrate Diet in treating active IBD. A prospective study of 142 people with IBD initiated on a Mediterranean diet demonstrated improvement in disease activity and quality of life [20]. A randomized trial comparing a specific carbohydrate diet to a Mediterranean diet in Crohn’s disease demonstrated no difference between the two diets in terms of symptomatic remission (~45%) and fecal calprotectin (~30–35%) response [21]. Additionally, researchers suggest that, based on its relative ease of following and additional health benefits, the Mediterranean diet may be a more practical option than the specific carbohydrate diet for most patients with mild to moderate Crohn’s disease [21]. Moreover, almost 50% of the participants experienced a clinical remission by 6 weeks of following the diets. Given that many dietary interventions have shown significant positive outcomes, including reduced gastrointestinal symptoms, reduced inflammation and improved quality of life, within 6–12 weeks of dietary intervention [21–25], a period of 8 weeks can be considered ideal to test in a pilot.

The recent American Association of Gastroenterology (AGA) 2024 guidelines provide specific recommendations for all IBD patients without contraindications to follow a Mediterranean eating pattern low in ultra processed foods [14]. Implementing these recommendations into practice can be challenging in settings without access to a dietician. Although gastroenterologists (GIs) may be aware and able to advise patients on the type of diet, few would have the expertise or time to provide specific details of the foods that are allowed or excluded in these diets. Published literature suggests significant gaps in knowledge relating to nutrition in IBD exist among health care providers. A survey of 223 providers demonstrated that only 51% of GIs rated their knowledge of nutrition in IBD as “very good” and 33% reported they did not routinely screen for malnutrition [26].

While apps for managing diet and lifestyle in IBD are becoming more common, few effectively support self-management or formally assess effectiveness in changing behavior or health outcomes. A recent review of available mobile health apps for IBD found that many IBD apps were designed for virtual care led by healthcare providers [27]. The study evaluated apps based on features like dietary support, goal setting, and behavior-change facilitation, highlighting apps such as LyfeMD [28], My IBD Care: Crohn’s and Colitis [29], MyGut App [30], Colitis Diary [31] and Crohn’s Diary [32]. Some apps were purely for tracking without educational information, and their effectiveness in improving behaviors, disease outcomes, or quality of life remains unclear. It was also unclear if there was patient engagement or input in the development of these apps. Although validated rating scales such as MARS (Mobile Application Rating Scale) [33] are often used to evaluate the quality of an app including engagement, functionality, aesthetic and information, it is not clear that these factors contribute to meaningful clinical outcomes. Therefore, we developed a nutrition guidance app (MyIBDDiet), co-designed with insights from patient research partners, to support patients living with IBD to self-manage and adopt a Mediterranean diet eating pattern.

## Objectives

This pilot study is designed to explore a structured approach to the evaluation of a mobile health app to empower self-management for people living with IBD. The primary study objectives are to evaluate the usability and acceptability of the MyIBDDiet app. The secondary objectives include evaluating the efficacy of the app in improving diet quality, diet knowledge, disease control, safety and quality of life.

## Materials and methods

### App development and device feasibility

We have co-developed a diet guidance and tracking app (MyIBDDiet) with patient research partners with the aim to improve diet quality and reduce inflammation. Features of the app allow for tracking of food intake using the Canadian Nutrient File (CNF) [34], and foods from the USDA (United States Department of Agriculture’s Food Data Central database) [35], CINE arctic nutrient file (Centre for Indigenous People’s Nutrition and Environment database) [36], INDB (Indian Nutrient Databank) [37], and OFF (Open Food Facts) [38] databases. It also tracks symptoms allowing users to correlate food intake with symptoms. The app provides immediate messaging feedback on the level of food processing [using NOVA classification [39]] and alignment of the participant’s food intake to an anti-inflammatory mediterranean-style eating pattern. A barcode scanner based on API food database helps users to identify foods with additives or emulsifiers that have been linked to IBD. Additionally, electronic resources and videos provide education on an anti-inflammatory pattern of eating, fiber and the intestinal microbiome, food additives and emulsifiers and practical substitutions in a restaurant.

As part of the app development process, device feasibility testing was conducted on version 1 of the app by academic researchers and patient research partners. Feedback primarily related to content clarity and interpretation as well as missing food categories and items, which led to incorporation of the Canadian Nutrition file database with more than 5000 foods along with additional foods from the USDA, CINE, INDB, and OFF databases expanding the available food choices to include branded foods and culturally appropriate foods. We aim to further expand the database based on the reporting of missing foods so that the database is oriented to include all the foods consumed commonly in the multi-cultural North American region. Moreover, clearer presentation and placement of information on food tags such as food processing level and mediterranean style eating pattern in the app were highlighted as areas for improvement. Foods were subsequently tagged into appropriate processing categories and Mediterranean-like eating tags by an experienced IBD dietitian. Additional feedback focused on interface layout and technical issues, including difficulties with video loading, symptom logging and app login functionality. These issues were resolved in a timely manner which provided us with an improved version 2 (V2) of the app suitable for use in the present study.

### Trial design and setting

**Fig 1:**
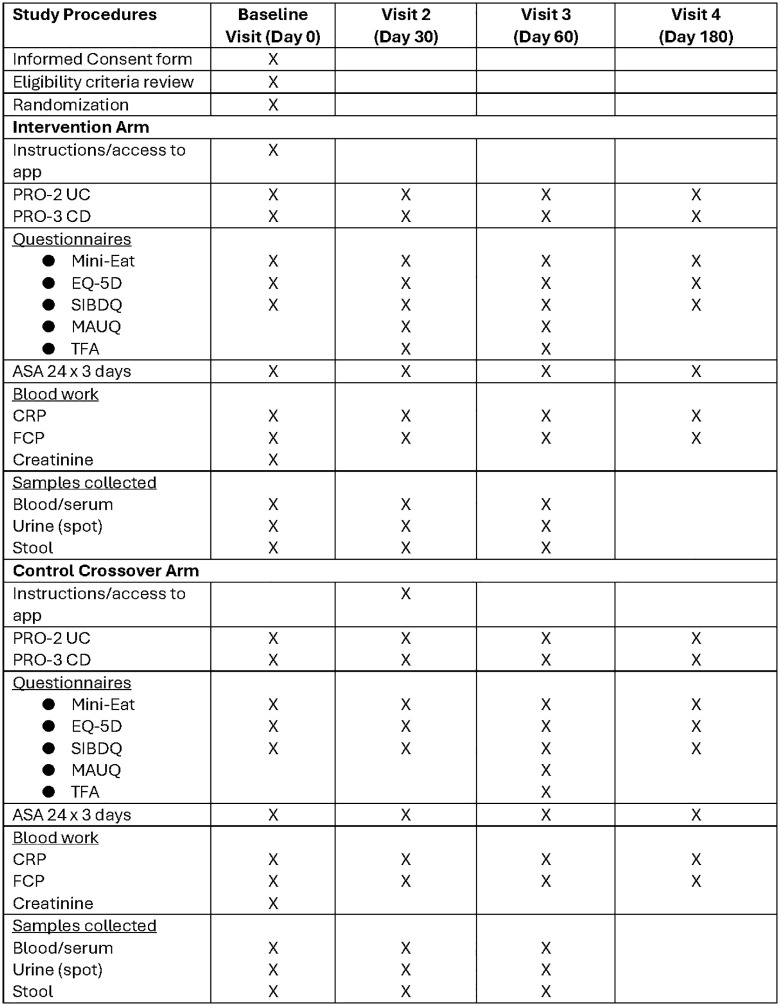
SPIRIT schedule of enrollment, interventions, and assessments. PRO-2 UC = Patient-Reported Outcome for ulcerative colitis, PRO-3 CD = Patient-Reported Outcome for Crohn’s disease, Mini-EAT = Mini-Eating Assessment Tool, EQ-5D = EuroQol, SIBDQ = Short Inflammatory Bowel Disease Questionnaire, MAUQ = mHealth App Usability Questionnaire, TFA = Theoretical Framework of Acceptibility, ASA24^®^ = Automated Self-Administered 24-Hour Dietary Assessment Tool, CRP = C-reactive protein, FCP = fecal calprotectin.

**Fig 2:**
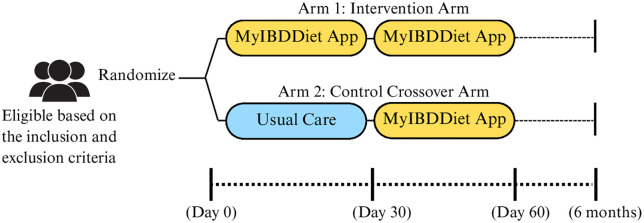
Overview of the Study Design.

This protocol was developed in accordance with the SPIRIT 2013 statement (S1 File). Fig 1 shows the SPIRIT schedule of enrollment, interventions and assessments. This single-centre, open-label pilot randomized trial will be conducted at the University of Alberta hospital, a tertiary care hospital located in Edmonton, Alberta. Participants will be randomized in a 1:1 ratio to either the Intervention Arm (receiving MyIBDDiet app for the entire duration of the study, i.e., 60 days) or the Control Crossover Arm (receiving usual care as per treating physician for the first 30 days followed by MyIBDDiet App for the latter half of the study) (Fig 2). Outcomes of interest will be assessed at baseline, 1 month, 2 months, and at 6 months follow-up. The 6-month timepoint will allow for assessment of persistence of behavioral and diet quality changes.

### Sample size

We aim to recruit 40 people living with IBD (20 in each arm). As this is a pilot feasibility and usability study, a formal power calculation was not done. The sample size of n = 40 (n = 20 each arm) is based on established recommendations for pilot trials, where it has been suggested that a minimum of 12–30 participants per arm are sufficient to estimate the variance required for a future power calculation [40,41]. The results of this pilot study will inform the sample size calculation for a future larger, multi-centre randomized trial to evaluate efficacy of MyIBDDiet App. Participants will be recruited for semi-structured interviews until thematic saturation is reached.

### Risk mitigation plan

The IBD Clinic at the University of Albertan actively follows approximately 5100 patients with IBD across a team of seven dedicated IBD physicians, representing a substantial and stable patient population to support recruitment for this trial. To further mitigate the risk of slower than anticipated recruitment, we will implement supplementary recruitment strategies, including distributing ethics approved recruitment materials through ethics approved social media platforms and University of Alberta newsletters. This has been a successful recruitment strategy for our previous studies.

### Eligibility criteria

The inclusion and exclusion criteria are outlined as below.

**Inclusion Criteria:**

1. Age ≥ 18 years
2. Able to provide informed consent
3. Established diagnosis of IBD determined by treating physician
4. Not in acute flare (inability to tolerate fibre diet)
5. Willing and able to adhere with all required study procedures
6. Able to read and speak English

**Exclusion Criteria:**

1. Short bowel syndrome
2. High ostomy output
3. Intestinal strictures
4. Pregnancy or breastfeeding
5. Malnutrition [screened by Canadian Nutrition Screening Tool (CNST)]
6. Not willing or able to comply with all required study procedures
7. Conditions requiring dietary restrictions (e.g., celiac disease, kidney disease, diabetes, eosinophilic esophagitis
8. Have other conditions that may require low fibre diet such as irritable bowel syndrome, gastroparesis
9. Currently on a therapeutic diet for IBD or using diet tool for IBD (e.g., Nutrition or diet Apps, specific carbohydrate diet, EEN, CDED, Mediterranean or anti-inflammatory diet)
10. Active malignancy

### Criteria for discontinuation

Participants can withdraw from the trial at any time at participant’s request, if there are adverse effects or worsening of IBD

### Enrollment and randomization

Potential participants, who may have been referred to this study through their primary care physician/gastroenterologist or self-referred by responding to recruitment posters, will be given a copy of the informed consent form (ICF) for review at their convenience. At a scheduled call or in-person visit, a member of the study team will explain the study in detail, answer any potential questions and obtain written informed consent from those who agree to take part in the study. Once informed consent is received, eligibility will be confirmed by accessing the medical charts for determining alignment with the eligibility criteria. Eligible participants will be enrolled in the study, scheduled for their baseline visit and randomized in a 1:1 ratio to either of the two study arms: Early Intervention Arm and Delayed Intervention Arm. Randomization will be performed using Research Electronic Data Capture (REDCap) [42] software hosted at the University of Alberta.

### Intervention arms and study procedures

#### Study arms.

**Fig 3:**
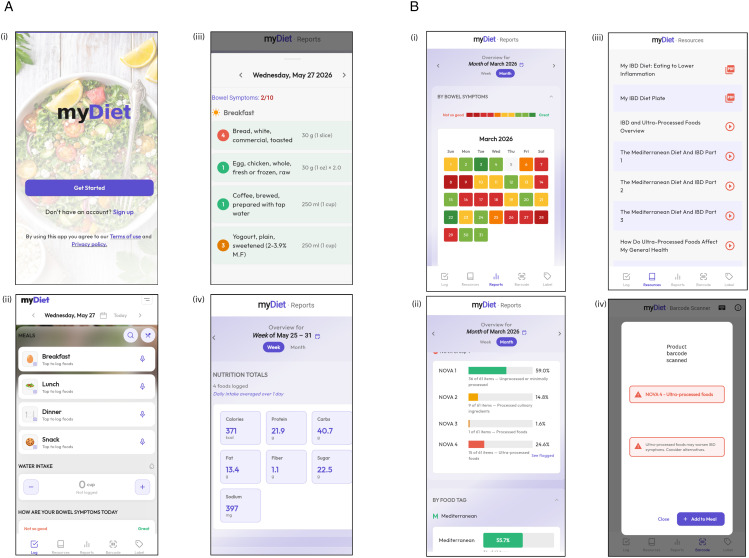
Snapshots of MyIBDDiet App illustrating various features of the app. (A) MyIBDDiet App is a nutrition education app co-developed by academic researchers and patient research partners. This figure shows snapshots of app features, explained as follows: (i) MyIBDDiet App landing and login screen (ii) MyIBDDiet App dashboard shows the food log, visual analogue scale for tracking bowel symptoms and taking notes (iii) shows food log with number tagging for level of processing where four indicates ultra-processed and one indicates no/minimal processing (iv) shows the nutritional information including caloric intake, protein intake, fibre and sodium intake amongst other parameters. Reprinted from https://myibddiet.vercel.app/ under a CC BY license, with permission from Seca Innovations, original copyright [2026] Fig 3B: Snapshots of MyIBDDiet App illustrating various features of the app. (B) This figure shows snapshots of app features, explained as follows: (i) shows the reports section- where the calendar shows the severity of bowel symptoms on each day of the month (ii) shows the breakdown of food items according to the level of processing and alignment with Mediterranean style eating. This feature can be adjusted to see a long-term trend over a week or a month (iii) shows the resources and nutrition educational videos created on topics like ultra-processed foods, recognition and replacement and information on anti-inflammatory diets such as Mediterranean diet (iv) shows the use of the barcode scanner to identify additives and emulsifiers in foods. Reprinted from https://myibddiet.vercel.app/ under a CC BY license, with permission from Seca Innovations, original copyright [2026].

Intervention Arm: Participants randomized to the Early Intervention Arm will receive access to MyIBDDiet App (Fig 3A and 3B) for 60 days. Participants will have access to all the features of the app including diet and symptom tracking, information on level of food processing, adherence to mediterranean-style eating, ability to correlate diet with bowel symptom severity and access to nutrition education resources. Use of the app is self-directed and participants will be encouraged to engage with the features as needed to support self-management. They are not required to use the app on a daily basis or for a set period of time. App engagement metrics will be collected at the backend to assess utilization patterns.

Control Crossover Arm: Participants will continue on the usual care for the first half of the study (first 30 days). Usual care represents any dietary advice or recommendations that the treating gastroenterologist may or may not provide the patient as part of usual care or what patients may be using on their own. Currently there are no standardized resources at our center but patients can be referred to an IBD dietitian. Providers typically direct patients to the Crohn’s Colitis Canada website. For the latter half of the study, participants will crossover to the MyIBDDiet App.

### Study procedures

The research coordinator will conduct the study at the clinic in person. Study procedures are outlined below:

Participant characteristics: Baseline characteristics, such as demographics, details of IBD diagnosis, therapies and disease activity, will be obtained from the participant and by reviewing their medical charts at the baseline visit (Day 0).

Anthropometrics: Anthropometric measurements, including height, weight, waist circumference and waist-to-hip ratio will be recorded at each clinical visit (Baseline, Day 30 and Day 60).

Nutritionally relevant questionnaires: Participants will be asked to complete a series of nutritionally relevant questionnaires, including the ASA24^®^ Dietary Assessment Tool and the Mini-EAT™ diet quality questionnaire. These will be completed at baseline, day 30, day 60 and day 180.

- ASA24^®^: The ASA24^®^ is an electronic, self-automated, 24-hour dietary recall tool developed to assess dietary intake data. Using the Canadian version of the ASA24^®^ (ASA24-Canada-2018), each participant will log all self-reported food and beverage intake from a 24-hour period for two previous weekdays and one previous weekend day [43]. They will also have the option to complete ASA24 in an interviewer-led way where a member of the study team will collect the dietary recall data from the participant and log in ASA 24 on their behalf. The Healthy Eating Index Score (HEI) [44] and Mediterranean Diet Serving Score (MDSS) [45] will be calculated based on the food recall data obtained from ASA24.
- Mini-EAT™: 9-item Mini-EAT™ is a short diet quality screener which has been validated for use in clinical settings [46]. It is scored based on the frequency of consumption of fruits, vegetables, grains (whole and refined), legumes, seafood, dairy (low and high fat), sweets and sugary drinks [46].

Quality of Life questionnaires: Health related quality of life will be assessed by EQ-5D and SIBDQ questionnaires.

- EuroQoL-5 Dimensions (EQ-5D): EQ-5D assesses health related quality of life across five dimensions: mobility, self-care, usual activities, pain or discomfort and anxiety or depression [47]. Each dimension has five levels of severity [47]. The questionnaire also uses a visual analogue scale that allows participants to quantitatively describe their overall health on the day of completing the questionnaire [47].
- Short Inflammatory Bowel Disease Questionnaire (SIBDQ): SIBDQ is an IBD-specific, widely used and validated quality of life tool that features 10 questions across four domains: Bowel symptoms, Systemic symptoms, Emotional function and social function [48].

Usability and Acceptability questionnaires:

- mHealth App Usability Questionnaire (MAUQ): MAUQ is a validated tool used to assess the usability of mobile health applications from the user’s perspective. It evaluates usability across key domains, including overall participant satisfaction, system information arrangement and usefulness [49]. Domain-specific and overall scores will be calculated.
- Theoretical Framework of Acceptability (TFA): The acceptability of the intervention was assessed using the TFA questionnaire, structured to measure acceptability across the key constructs of affective attitude, burden, ethicality, intervention coherence, opportunity costs, perceived effectiveness and self-efficacy [50].

Semi-structured Interviews: Semi-structured interviews will be conducted virtually via an online conferencing platform. All the participants who initiated the intervention will be invited to participate in the interviews. The interview questions have been framed using the CFIR framework [51] and the focus of the interview is to identify barriers and facilitators to the use of MyIBDDiet App (S2 File). Interviews will be conducted by research team members experienced in conducting qualitative research. All identifying information will be removed and interviews will be transcribed verbatim.

Biological specimen collection and analytical procedures: C-reactive protein and fecal calprotectin levels will be collected for assessing disease activity as per standard of care. Urine sodium and chloride levels will be measured using a dipstick test to relate to processed foods consumption. Spot urine sodium has been used in studies to help validate processed food questionnaires [52]. We have also elected to use spot urine chloride as the availability of urine sodium strips are limited and urine chloride strips have been validated against 24-hour urine excretion and found to provide reasonable approximation [53]. Stool samples will be collected and processed for 16S rRNA sequencing to assess microbial composition. As the accuracy of diet questionnaires can be challenging in clinical trials, we will be collecting stool and serum samples to evaluate metabolite signatures to correlate with the diet questionnaire data [54]. We will perform untargeted metabolomic profiling using chemical isotope labelling liquid chromatography-mass spectrometry (CIL LC-MS).

### Primary, secondary and exploratory outcomes

The primary outcome of usability will be evaluated using a mixed-method quantitative [mHealth App Usability Questionnaire (MAUQ) and Theoretical Framework of Acceptability (TFA)] and qualitative (semi-structured interviews) approach.

Secondary outcomes of interest include clinical efficacy evaluated by change in diet quality [Mini-EAT questionnaire, Automated Self-administered 24-Hour Dietary Assessment Tool (ASA-24), Healthy Eating Index (HEI), Mediterranean Diet Serving Score (MDSS)], urine sodium and chloride (biomarkers of processed food intake), changes in disease activity [Patient Reported Outcome (PRO2 UC and PRO3 CD), C-reactive protein, fecal calprotectin] and changes in quality of life [EQ-5D, SIBDQ]. Behaviour changes and diet knowledge will be assessed objectively by measuring changes in diet quality scores, changes in the nutrient intake data obtained from ASA 24 and subjectively by assessing responses to individual questions on changes in diet in the semi-structured interview. To assess safety, systematic monitoring of adverse events and serious adverse events will be done at each time point and in between time points if symptoms worsen.

Exploratory outcomes include changes in fecal microbiome and serum and fecal metabolites to correlate with change in diet and disease activity.

### Data management

We will use REDCap hosted at the University of Alberta as a secure, web-based application for data collection and management in our study. The questionnaires for participants will be completed during the clinical visit with the assistance of the research team and then transferred into the REDCap system. All study personnel will undergo Good Clinical Practice (GCP) training and be informed of their responsibilities regarding participant privacy and confidentiality through detailed protocol discussions prior to participant recruitment. Only study personnel authorized by the principal investigator will have access to study materials and data.

### Ethical approval, trial status and timeline

This study will be conducted in accordance with Good Clinical Practice guidelines and has been approved by the University of Alberta Health Research Ethics Board (HREB Biomedical – Ethics ID: Pro00141681). Recruitment is anticipated to begin in March 2026 and to complete by March 2028. Data analysis is expected to be complete by August 2028. Manuscript writing and publication of results is expected by December 2028.

### Statistical analysis plan

The main analytical steps in this study include descriptive statistics, pre-post comparisons, and between-group comparison tests. All quantitative analyses will be conducted on an intention-to-treat (ITT) basis, including all participants who undergo randomization [55]. This pilot randomized study will be conducted over 60 days, comprising two 30-day periods of app use.

Primary and secondary quantitative outcomes: To account for the repeated measures (Baseline, Day 30, Day 60, Day 180) and the crossover study design, we will utilize Linear Mixed Models (LMM) [56,57]. This approach is preferred as it handles missing data robustly via Restricted Maximum Likelihood (REML) estimation and accounts for within- subject and between-subject variability. The model will include “Group”, “Time” and “Group X Time” interactions as fixed effects, with a random intercept for each participant. In the event of model non-convergence, the random effects structure will be simplified.

Handling of missing data: We anticipate an attrition rate of 10–15%. Linear Mixed Models allow for the inclusion of participants with missing timepoints under missing at random assumption. If missing data exceeds 20%, Multiple imputations by chained Equations (MICE) will be performed [58].

Effect size and Variance: As this is a pilot study, the focus of analysis is not solely on p-values, but on point estimates and confidence intervals. We will calculate Cohen’s d effect sizes for change in quantitative outcomes to provide the necessary variance and magnitude of effect estimates required for a power calculation in a future larger trial. We will be using SPSS version 29.0.2.0 (20) for statistical analyses.

Semi-structured interviews: The interviews will be transcribed verbatim. Thematic analysis will be done independently by two researchers using Nvivo which is a qualitative data analysis software and any conflicts will be resolved by a third reviewer who is a senior gastroenterologist and principal investigator [59].

### Microbial and metabolomic analysis

Microbial diversity will be evaluated through both α-diversity and β-diversity metrics, specifically using the Shannon index and Bray-Curtis dissimilarity, respectively. Metabolomics datasets will be analyzed using the KEGG database [60] to facilitate pathway mapping and biological interpretation.

Principal Coordinates Analysis (PCoA) will be employed to visualize and compare microbiome profiles pre- and post-intervention at baseline, day 30 and day 60, as well as between usual care and MyIBDDiet app groups at baseline and day 30. Univariate analysis, specifically ANOVA, will be applied to identify statistically significant features across groups. In addition, multivariate analysis via Principal Component Analysis (PCA) will be employed to visualize and compare metabolic profiles between pre- and post-intervention samples, as well as between subjects in usual care versus MyIBDDiet app group. As this is a pilot study with a small sample size, multivariable analyses and PCA will be strictly exploratory.

### Dissemination plans

Results of the study will be communicated by email/social media channels to participants after study closes and statistical analyses have been completed. Results from the study will be presented in conferences, uploaded on clinicaltrials.gov and communicated through preparing a manuscript and submitting for publication.

## Discussion

There is growing evidence to support the role of diet in the management of IBD and recent guidelines have recommended a Mediterranean diet and avoidance of ultra-processed foods for all patients without contraindications [14]. Knowledge dissemination and implementation of these recommendations may be challenging in the current healthcare delivery model. Digital health tools have the potential to help bridge health service delivery gaps however few mobile health apps for IBD empower self-management and or have been formally evaluated for effectiveness to change behaviours, alter disease course or improve quality of life (27).

Our research proposal is designed to explore a more structured approach to the development and evaluation of a mobile health app to empower self-management. Following the knowledge to action framework, we engaged patient partners early to conduct semi-structured interviews and focus groups to identify barriers to use. Patient partners were engaged in the co-design of the MyIBDDiet app prototype and will continue to be involved in the process of iterative refinement of the app. We purposefully selected a narrow focus for the app to educate and assist patients to adopt an anti-inflammatory diet as this allows for more precise measurements of clinical effectiveness and change in behavior than more comprehensive apps that combine all types of lifestyle interventions. The potential downside to comprehensive apps is that it may overwhelm patients, and not all users may be ready to alter multiple lifestyle factors at once. Our evaluation plan is more rigorous than other mobile apps. We have included not only usability but questionnaires that help determine change in diet quality (taking in less highly processed foods, adopting a healthier eating pattern), clinical disease measures (including objective markers) and quality of life. The pilot data generated may inform the design of a larger scale RCT and future mobile app development and evaluation studies.

Feedback on usability and acceptability through validated questionnaires and semi-structured interviews will be used to refine the app. In future studies, we may analyze the long-term effects and sustainability of MyIBDDiet by extending our data collection timeframe. We will also consider a larger group of participants as well as including participants from multiple centers within and outside the province of Alberta.

## Supporting information

S1 File Completed Standard Protocol Items: Recommended for Intervention trials (SPIRIT) checklist. (DOC)

S2 File Semi-structured interview script for the study. (DOCX)

## References

[1] McDowell C, Farooq U, Haseeb M. Inflammatory Bowel Disease. StatPearls. 2023. 29262182

[2] Coward S, Benchimol EI, Kuenzig ME, Windsor JW, Bernstein CN, Bitton A, et al. The 2023 Impact of Inflammatory Bowel Disease in Canada: Epidemiology of IBD. J Can Assoc Gastroenterol. 2023 Sep 1;6(Suppl 2):S9. doi: 10.1093/JCAG/GWAD004 37674492 PMC10478802

[3] Bueno-Hernández N, Yamamoto-Furusho JK, Mendoza-Martínez VM. Nutrition in inflammatory bowel disease: strategies to improve prognosis and new therapeutic approaches. Diseases. 2025;13(5):139. doi: 10.3390/DISEASES13050139 40422571 PMC12110586

[4] Jabłońska B, Mrowiec S. Nutritional Status and Its Detection in Patients with Inflammatory Bowel Diseases. Nutrients. 2023;15(8):1991. doi: 10.3390/NU15081991 37111210 PMC10143611

[5] Czuber-Dochan W, Morgan M, Hughes LD, Lomer MCE, Lindsay JO, Whelan K. Perceptions and psychosocial impact of food, nutrition, eating and drinking in people with inflammatory bowel disease: a qualitative investigation of food-related quality of life. J Hum Nutr Diet. 2020;33(1):115–27. doi: 10.1111/JHN.12668 31131484

[6] Godala M, Gaszyńska E, Durko Ł, Małecka-Wojciesko E. Dietary Behaviors and Beliefs in Patients with Inflammatory Bowel Disease. J Clin Med. 2023;12(10):3455. doi: 10.3390/jcm12103455 37240560 PMC10219397

[7] Bergeron F, Bouin M, D’Aoust L, Lemoyne M, Presse N. Food avoidance in patients with inflammatory bowel disease: what, when and who?. Clinical Nutrition. 2018;37(3):884–9. doi: 10.1016/J.CLNU.2017.03.010 28359542

[8] Day AS, Yao CK, Costello SP, Andrews JM, Bryant RV. Food‐related quality of life in adults with inflammatory bowel disease is associated with restrictive eating behaviour, disease activity and surgery: A prospective multicentre observational study. J Human Nutrition Diet. 2021;35(1):234–44. doi: 10.1111/jhn.12920 34008222

[9] Yelencich E, Truong E, Widaman AM, Pignotti G, Yang L, Jeon Y, et al. Avoidant Restrictive Food Intake Disorder Prevalent Among Patients With Inflammatory Bowel Disease. Clinical Gastroenterology and Hepatology. 2022;20(6):1282-1289.e1. doi: 10.1016/j.cgh.2021.08.009 34389486

[10] Noejovich CV, Miranda P, Rueda GH, Yuan Y, Szeto J, Patel R, et al. Understanding dietary beliefs, behaviors, and barriers in inflammatory bowel disease: A scoping review. Clin Nutr ESPEN. 2026;71:102818. doi: 10.1016/j.clnesp.2025.11.133 41338453

[11] Goh J, O’Morain CA. Review article: nutrition and adult inflammatory bowel disease. Aliment Pharmacol Ther. 2003;17(3):307–20. doi: 10.1046/j.1365-2036.2003.01482.x 12562443

[12] Lucendo AJ, De Rezende LC. Importance of nutrition in inflammatory bowel disease. World J Gastroenterol. 2009;15(17):2081–8. doi: 10.3748/wjg.15.2081 19418580 PMC2678578

[13] Gold SL, Raman M. Malnutrition assessment in patients with inflammatory bowel disease. Can IBD Today. 2023. doi: 10.58931/cibdt.2023.119

[14] Hashash JG, Elkins J, Lewis JD, Binion DG. AGA Clinical Practice Update on Diet and Nutritional Therapies in Patients With Inflammatory Bowel Disease: Expert Review. Gastroenterology. 2024;166(3):521–32. doi: 10.1053/j.gastro.2023.11.303 38276922

[15] Lo CH, Khandpur N, Rossato SL, Lochhead P, Lopes EW, Burke KE, et al. Ultra-processed foods and risk of Crohn’s disease and ulcerative colitis: a prospective cohort study. Clin Gastroenterol Hepatol. 2021;20(6):e1323. doi: 10.1016/J.CGH.2021.08.031 34461300 PMC8882700

[16] Narula N, Wong ECL, Dehghan M, Mente A, Rangarajan S, Lanas F, et al. Association of ultra-processed food intake with risk of inflammatory bowel disease: prospective cohort study. BMJ. 2021;374:1554. doi: 10.1136/BMJ.N1554 34261638 PMC8279036

[17] Chen J, Wellens J, Kalla R, Fu T, Deng M, Zhang H, et al. Intake of Ultra-processed Foods Is Associated with an Increased Risk of Crohn’s Disease: A Cross-sectional and Prospective Analysis of 187 154 Participants in the UK Biobank. J Crohns Colitis. 2023;17(4):535–52. doi: 10.1093/ecco-jcc/jjac167 36305857 PMC10115229

[18] Kakkadasam Ramaswamy P, Gold Coast Inflammatory Bowel Diseases Research Group. Exclusive enteral nutrition with oral polymeric diet helps in inducing clinical and biochemical remission in adults with active Crohn’s disease. JPEN J Parenter Enteral Nutr. 2022;46(2):423–32. doi: 10.1002/jpen.2273 34618355

[19] Yanai H, Levine A, Hirsch A, Boneh RS, Kopylov U, Eran HB, et al. The Crohn’s disease exclusion diet for induction and maintenance of remission in adults with mild-to-moderate Crohn’s disease (CDED-AD): an open-label, pilot, randomised trial. Lancet Gastroenterol Hepatol. 2022;7(1):49–59. doi: 10.1016/S2468-1253(21)00299-5 34739863

[20] Chicco F, Magrì S, Cingolani A, Paduano D, Pesenti M, Zara F. Multidimensional impact of Mediterranean diet on IBD patients. Inflamm Bowel Dis. 2020;27(1):1. doi: 10.1093/IBD/IZAA097 32440680 PMC7737160

[21] Lewis JD, Sandler RS, Brotherton C, Brensinger C, Li H, Kappelman MD, et al. A Randomized Trial Comparing the Specific Carbohydrate Diet to a Mediterranean Diet in Adults With Crohn’s Disease. Gastroenterology. 2021;161(3):837-852.e9. doi: 10.1053/j.gastro.2021.05.047 34052278 PMC8396394

[22] Godala M, Gaszyńska E, Zatorski H, Małecka-Wojciesko E. Dietary Interventions in Inflammatory Bowel Disease. Nutrients. 2022;14(20):4261. doi: 10.3390/nu14204261 36296945 PMC9607252

[23] Pedersen N, Ankersen DV, Felding M, Wachmann H, Végh Z, Molzen L, et al. Low-FODMAP diet reduces irritable bowel symptoms in patients with inflammatory bowel disease. World J Gastroenterol. 2017;23(18):3356–66. doi: 10.3748/wjg.v23.i18.3356 28566897 PMC5434443

[24] Olendzki BC, Silverstein TD, Persuitte GM, Ma Y, Baldwin KR, Cave D. An anti-inflammatory diet as treatment for inflammatory bowel disease: a case series report. Nutr J. 2014;13:5. doi: 10.1186/1475-2891-13-5 24428901 PMC3896778

[25] Bodini G, Zanella C, Crespi M, Lo Pumo S, Demarzo MG, Savarino E, et al. A randomized, 6-wk trial of a low FODMAP diet in patients with inflammatory bowel disease. Nutrition. 2019;:67-68:110542. doi: 10.1016/J.NUT.2019.06.023 31470260

[26] Tinsley A, Ehrlich OG, Hwang C, Issokson K, Zapala S, Weaver A, et al. Knowledge, Attitudes, and Beliefs Regarding the Role of Nutrition in IBD Among Patients and Providers. Inflamm Bowel Dis. 2016;22(10):2474–81. doi: 10.1097/MIB.0000000000000901 27598738

[27] Gold SL, Chiew BA, Rajagopalan V, Lavallee CM. Identification and evaluation of mobile applications for self-management of diet and lifestyle for patients with inflammatory bowel disease. J Can Assoc Gastroenterol. 2023;6(5):186. doi: 10.1093/JCAG/GWAD029 37811532 PMC10558196

[28] lyfemd. https://lyfemd.com/ 2026 February 17.

[29] My IBD Care: Crohn’s or Colitis Management App. https://ampersandhealth.co.uk/myibdcare/ 2026 February 17.

[30] MyGut App - Crohn’s and Colitis Canada. https://crohnsandcolitis.ca/Support-for-You/MyGut 2026 February 17.

[31] Colitis Diary 3 App - App Store. https://apps.apple.com/ca/app/colitis-diary-3/id6463022087 2026 February 17.

[32] Crohn’s Diary 3 App - App Store. https://apps.apple.com/ca/app/crohns-diary-3/id6463499470 2026 February 17.

[33] Mobile Application Rating Scale (MARS) App Classification.

[34] The Canadian Nutrient File. https://www.canada.ca/en/health-canada/services/food-nutrition/healthy-eating/nutrient-data/canadian-nutrient-file-about-us.html 2026 January 22.

[35] USDA FoodData Central. https://fdc.nal.usda.gov/ 2026 May 31.

[36] CINE’s Arctic Nutrient File. Centre for Indigenous Peoples’ Nutrition and Environment - McGill University. https://www.mcgill.ca/cine/resources/nutrient 2026 May 31.

[37] Indian Nutrient Databank (INDB) - Anuvaad Solutions. https://www.anuvaad.org.in/indian-nutrient-databank/ 2026 May 31.

[38] Home | Open Food Facts - Connect. https://connect.openfoodfacts.org/ 2026 May 31.

[39] Monteiro CA, Cannon G, Moubarac J-C, Levy RB, Louzada MLC, Jaime PC. The UN Decade of Nutrition, the NOVA food classification and the trouble with ultra-processing. Public Health Nutr. 2018;21(1):5–17. doi: 10.1017/S1368980017000234 28322183 PMC10261019

[40] Julious SA. Sample size of 12 per group rule of thumb for a pilot study. Pharm Stat. 2005;4(4):287–91. doi: 10.1002/PST.185

[41] Hertzog MA. Considerations in determining sample size for pilot studies. Res Nurs Health. 2008;31(2):180–91. doi: 10.1002/nur.20247 18183564

[42] REDCap [Internet]. cited 2026 Feb 17. https://project-redcap.org/

[43] ASA24® Dietary Assessment Tool. https://epi.grants.cancer.gov/asa24/ 2026 January 22.

[44] Steps for Calculating Healthy Eating Index Scores. https://epi.grants.cancer.gov/hei/calculating-hei-scores.html 2026 January 22.

[45] Monteagudo C, Mariscal-Arcas M, Rivas A, Lorenzo-Tovar ML, Tur JA, Olea-Serrano F. Proposal of a Mediterranean Diet Serving Score. PLoS One. 2015;10(6):e0128594. doi: 10.1371/journal.pone.0128594 26035442 PMC4452755

[46] Lara-Breitinger KM, Inojosa JRM, Li Z, Kunzova S, Lerman A, Kopecky SL. Validation of a Brief Dietary Questionnaire for Use in Clinical Practice: Mini‐EAT (Eating Assessment Tool). Journal of the American Heart Association: Cardiovascular and Cerebrovascular Disease. 2022;12(1):e025064. doi: 10.1161/JAHA.121.025064 36583423 PMC9973598

[47] Herdman M, Gudex C, Lloyd A, Janssen M, Kind P, Parkin D, et al. Development and preliminary testing of the new five-level version of EQ-5D (EQ-5D-5L). Qual Life Res. 2011;20(10):1727–36. doi: 10.1007/s11136-011-9903-x 21479777 PMC3220807

[48] Irvine EJ, Zhou Q, Thompson AK. The Short Inflammatory Bowel Disease Questionnaire: A Quality of Life Instrument for Community Physicians Managing Inflammatory Bowel Disease. Am J Gastroenterol. 1996;91(8):1571–8. 8759664

[49] Zhou L, Bao J, Setiawan IMA, Saptono A, Parmanto B. The mHealth App Usability Questionnaire (MAUQ): Development and Validation Study. JMIR Mhealth Uhealth. 2019;7(4):e11500. doi: 10.2196/11500 30973342 PMC6482399

[50] Sekhon M, Cartwright M, Francis JJ. Acceptability of healthcare interventions: an overview of reviews and development of a theoretical framework. BMC Health Serv Res. 2017;17(1):1–13. doi: 10.1186/S12913-017-2031-8 28126032 PMC5267473

[51] Damschroder LJ, Aron DC, Keith RE, Kirsh SR, Alexander JA, Lowery JC. Fostering implementation of health services research findings into practice: a consolidated framework for advancing implementation science. Implementation Science. 2009;4(1):1–15. doi: 10.1186/1748-5908-4-50 19664226 PMC2736161

[52] Sarbagili-Shabat C, Zelber-Sagi S, Fliss Isakov N, Ron Y, Hirsch A, Maharshak N. Development and validation of processed foods questionnaire (PFQ) in adult inflammatory bowel diseases patients. Eur J Clin Nutr. 2020;74(12):1653–60. doi: 10.1038/s41430-020-0632-5 32322049

[53] Heeney ND, Lee RH, Hockin BCD, Clarke DC, Sanatani S, Armstrong K, et al. At-home determination of 24-h urine sodium excretion: Validation of chloride test strips and multiple spot samples. Auton Neurosci. 2021;233:102797. doi: 10.1016/j.autneu.2021.102797 33773398

[54] Guasch-Ferré M, Bhupathiraju SN, Hu FB. Use of Metabolomics in Improving Assessment of Dietary Intake. Clin Chem. 2018;64(1):82–98. doi: 10.1373/clinchem.2017.272344 29038146 PMC5975233

[55] McCoy CE. Understanding the Intention-to-treat Principle in Randomized Controlled Trials. West J Emerg Med. 2017;18(6):1075–8. doi: 10.5811/westjem.2017.8.35985 29085540 PMC5654877

[56] Laird NM, Ware JH. Random-effects models for longitudinal data. Biometrics. 1982;38(4):963–74. doi: 10.2307/2529876 7168798

[57] Molenberghs G, Verbeke G. Linear Mixed Models for Longitudinal Data. Springer Series in Statistics. 2000. 10.1007/978-1-4419-0300-6

[58] van Buuren CGM, Oudshoorn S, van Buuren S, Oudshoorn C. Multivariate Imputation by Chained Equations MICE V1.0 User’s Manual. TNO Prevention and Health. 2000.

[59] Braun V, Clarke V. Using thematic analysis in psychology. Qual Res Psychol. 2006;3(2):77–101. doi: 10.1191/1478088706QP063OA

[60] Kanehisa M, Furumichi M, Sato Y, Kawashima M, Ishiguro-Watanabe M. KEGG for taxonomy-based analysis of pathways and genomes. Nucleic Acids Res. 2023;51(D1):D587–92. doi: 10.1093/nar/gkac963 36300620 PMC9825424

