# Supplementary material for: A pilot randomized trial on the usability and acceptability of an app (MyIBDDiet) to improve the self-management of anti-inflammatory diet for individuals with inflammatory bowel disease: A protocol paper
Source: PLoS One. 2026 Jul 2;21(7):e0353123. doi: 10.1371/journal.pone.0353123 (PMC13327250; doi:10.1371/journal.pone.0353123)
Supplement: S2 File — (DOCX) [file pone.0353123.s002.docx]

**S2 File: Semi-structured interview script for the study**

Semi-structured Interview Guide: Semi-structured Interviews to assess barriers and facilitators to the usability and acceptability of MyIBDDiet App in improving the self-management of anti-inflammatory diet for individuals with inflammatory bowel disease.

Study ID:__________

Participant ID:__________

Interviewer Name: ____________

Interview Date:__________

The purpose of this interview is to assess the barriers and facilitators to the usability and acceptability of MyIBDDiet App focused on improving nutrition education for people with IBD.

1. **Introduction**

*Greet the patient and thank him/her for giving an appointment for an interview.*

*Introduce yourself (interviewer), if the interviewee is not already familiar with you.*

*Provide the interviewee with the leaflet on the study design and briefly explain about the study.*

*Briefly explain the goal of MyIBDDiet App and the focus of this interview to gain insights into the barriers and facilitators of using the app.*

*Explain about confidentiality and use of the study outcomes. Introduce the consent form.*

*Ask for consent to video recording and note taking.*

1. **Interview**
2. **Nutrition and IBD**

*The purpose of this section is to help relax the interviewee and expand on their nutritional concerns.*

1. What role do you think nutrition plays in managing your IBD?
2. What strategies have you previously used to self-manage the nutritional aspect of IBD? *Which of these worked for you and why? Which did not work and why?*
3. Have you consulted a dietitian for nutritional advice in the past? If yes, how has this helped you with managing your diet and symptoms?
4. Have you used any nutrition-related digital tools in the past? If yes, how has this helped you with managing your diet and symptoms?
5. **Thoughts on the usability and effectiveness of MyIBDDiet App**

*The purpose of this section is to understand the interviewee’s thoughts about the usability and effectiveness of MyIBDDiet App. Identify potential challenges to the use of the app, and gain insights on the potential facilitators to the use of the app.*

1. What are your overall impressions of MyIBDDiet App?

*For example: What do you think about the design or layout of the app, is the information relevant and adequate, is the level of detail or complexity of the information appropriate, is the information easily accessible, how did logging and tracking work for you, is the app user friendly and intuitive to use?*

1. What features of the app you found most useful? *Why did you find this feature useful? How did this feature help you with managing your diet or symptoms?*
2. What are your thoughts on symptom tracking and the ability to correlate it with your meals? *Did this feature help with self-management of your symptoms? If yes, how? If not, why do you think it did not work for you? How can this be improved?*
3. How relevant did you find the recommendations and information provided for your condition and symptoms?
4. How has using the app influenced your nutrition knowledge and understanding?
5. How has self management of diet and symptoms changed since you started using the app? *Can you give some examples? What made you make these changes?*
6. What did you find lacking in terms of information available in the app? *How do you think that information will help you?*
7. Given that this version of the app is a prototype, we are aware that it needs improvements on the technical front. However, we would like your thoughts on the technological aspects of using the app?
8. What future improvements would you recommend for the app? *What features would you like to see included in the app? Why do you think they will be useful?*
9. **Thoughts on the influence of external and internal factors on the usage of app**

*This section will focus on understanding the perspectives around the support available to use the app in the social settings including the healthcare environment, interviewee’s social settings, family and personal space.*

1. Did the education or information provided in the app align with advice you have received from healthcare providers? *Please elaborate with examples.*
2. How easy or difficult was it for you to use the app and fit it into your daily routine*? At what times or in which situations were you most likely to use the app? Were there any situations where you wanted to use it but could not?*
3. What challenges did you experience in incorporating this tool into your daily life for self-management?
4. Did you receive support from family members and healthcare professionals regarding app use? What were their views on the app?
5. **Thoughts on readiness to adopt**

*The purpose of these questions is to assess the readiness of the interviewee to utilise the app and the information obtained from it in self-managing diet and symptoms in everyday life.*

1. How confident did you feel in managing your nutritional needs before using the app compared with after using it?
2. In your opinion, how long would someone need to use the app to feel confident and sufficiently informed to manage their nutrition on their own? *Do you think using the app requires ongoing engagement, or is it something that is most helpful with more intensive use initially and then minimal use over time? Please explain.*
3. **Closing**
4. Is there anything else you would like to share about your experience with the handout?

*Thank interviewee for time and inputs. Assure the sharing of study results with interviewees. Ask permission to get back to the interviewee for any clarifications/further information.*

*NOTE: The questions are subject to be revised based on the insights gained from the initial interviews.*
