# Supplementary material for: A pilot randomized trial on the usability and acceptability of an app (MyIBDDiet) to improve the self-management of anti-inflammatory diet for individuals with inflammatory bowel disease: A protocol paper
Source: PLoS One. 2026 Jul 2;21(7):e0353123. doi: 10.1371/journal.pone.0353123 (PMC13327250; doi:10.1371/journal.pone.0353123)
Supplement: Attachment — Submitted filename: Response to Reviewers_MyIBDDiet Protocol.docx [file pone.0353123.s003.docx]

**Response to Reviewers**

**Editor comments:**

1. It is necessary to establish a period of time that reflects real changes proposed in the protocol; 30- 60 days could be few time, but please reference the period selected by you.

Response: We appreciate the reviewer’s feedback regarding the study duration. We selected the 60-day intervention period for several strategic reasons. Previous research on dietary interventions in IBD indicates that significant improvements in diet quality, inflammatory markers, and patient-reported outcomes can be typically observed within a 4- to 8-week window (Lewis et al., 2021; Manski et al., 2024). Additionally, as this is a pilot study, a 60-day timeframe is appropriate to assess core app engagement and "proof of concept" for behavioral change before committing to a longer-term definitive trial. Also, we want to ensure participants use the app on a regular, high-frequency basis. The 2-month duration was chosen to maximize consistent adherence, mitigating the risk of "engagement fatigue" often seen in longer digital health interventions. The protocol includes a 6-month follow-up period which we think help address sustained use or knowledge retention. This will allow us to evaluate long-term retention and the frequency of app use once the active trial phase has ended. The rationale has been updated in the manuscript as suggested (Lines 77-88)

2. Clearly describe how you will measure behavioral changes, diet knowledge and safety of the app.

Response: Thank you for this comment. It has been clarified as below:

Behavioral Changes: To capture a holistic view of behavior change and the resulting dietary shifts, we will use a mixed-methods approach. Objective changes will be assessed using the diet quality questionnaire (Mini-EAT) and changes in the nutrient intake data generated via ASA24 as well as the app backend data showing food tracking over time. Subjective changes will be captured through semi-structured interviews, allowing participants to describe the lived experience of altering food choices.

Dietary Knowledge: As there is currently no single validated questionnaire for testing specific dietary knowledge in this context (in particular for ultra-processed foods), we evaluate knowledge through patterns of change mainly the translation of knowledge into action. As having knowledge versus putting it to application are two different concepts and it is important for us to measure if the knowledge gained is being put to action. Therefore, we will measure it via changes in eating habits and also track changes in ultra-processed food intake.

Safety of using the app: There will be continuous monitoring and reporting of all adverse events (AEs) and serious adverse events (SAEs) throughout the study duration. Moreover, to ensure participant safety, individuals experiencing an active flare, having a bowel obstruction or a high risk of bowel obstruction related to dietary fiber changes have been excluded from the study protocol.

Updated in the manuscript as suggested (Lines 348-352)

3. More references are needed to explain the eating behavior changes related to disease activity.

Response: More references have been added to explain the eating behaviour changes related to disease activity (Lines 63-65)

4. Suggest this study as a exploratory in order to respond the primary objective and a randomized trial to evaluate diet quality, disease control and quality of life.

Response: We appreciate the reviewer’s perspective on the study's trajectory. We agree that a large-scale randomized controlled trial (RCT) is the gold standard for evaluating clinical outcomes such as diet quality, disease control, and quality of life. However, we believe the current study is a critical exploratory and foundational step. While the app was developed by academic researchers and patient partners who have conducted initial usability testing, it has not yet been deployed within a diverse, larger-scale population. Our primary objective at this stage is to integrate lived experience and optimize functionality. Following the successful completion of this exploratory phase and the refinement of the app based on participant feedback, we intend to conduct a future randomized trial with the primary aim to rigorously evaluate its effectiveness regarding clinical outcomes and quality of life.

5. Describe if other anthropometric measurements than height and weight will be recorded.

Response: We thank the reviewer for bringing this important point to light. We have decided to expand the anthropometrics methodology to include waist circumference and waist-to-hip ratio in the measurements, along with height and weight. However, I would like to bring to your attention that the primary goal of the app is not weight loss, but rather to focus on eating overall healthier foods. We have not anticipated using it for weight loss at this stage. In the future, as we expand the study, we may possibly include body composition parameters using ultrasonography or existing MRI and CT scans, but for now, we want to stay focused on the primary goal of healthy eating patterns.

Information updated in manuscript (Lines 280-281)

Please indicate why height need to be measured on each visit, do you expect any change?

Response: Thank you for your insightful comment. We agree height is not expected to change during the study in the population, we have revised the protocol to reflect the height will only be measured at baseline.

6. Consider RM ANOVA to test changes over the time.

Response: Thank you for the useful suggestion. We have updated our statistical plan based on feedback received from reviewer 1 and decided to use Linear Mixed-effects Model (LMM). As LMM can also handle missing data and accounts for within-subject correlation across multiple timepoints, we have decided to switch to this. Updated statistical analyses strategy in manuscript (Lines 370-437)

**Reviewer 1:**

**Major**

1) the is no mention of the CONSORT statement.

Response: Thank you for this observation. We have followed the SPIRIT (Standard Protocol Items: Recommendations for Interventional Trials) statement which is internationally recognized guideline specifically designed for the reporting of study protocols. Since we are only reporting the study design and the protocol in this manuscript, we decided to report the SPIRIT checklist and will be reporting the CONSORT checklist once we publish the results and outcomes.

2) in the methods section I expected a plan to deal with missing data. are any expected? if yes are multiple imputation approaches considered?

Response: Thank you for your feedback. We have clarified the plan for missing data in the revised manuscript. We will utilize the restricted maximum likelihood approach of Linear Mixed Models (LMM) to handle the missing data. However, if missing data exceeds 20%, we will implement Multiple Imputation by Chained Equations (MICE) to ensure robustness. Updated in manuscript (Lines 378-388)

3) it was not clear to me what the multivariable analyses will add with a sample of this size. identifying predictors in a sample of 40, even as a secondary set of outcomes, is problematic. similarly, about the PCA. if the authors need to keep those analyses, they need to explain they are strictly exploratory.

Response: This is an excellent point. We have revised the manuscript to clearly state that the multivariable predictors and PCA will be strictly exploratory. The purpose of these analyses will be to generate hypothesis to be tested in future larger trials. Updated in manuscript (Lines 436-437)

4) the main analysis, the comparison of the two group, is planned using a test that only reports statistical significance - how will that inform a future study? the authors need to obtain a measure of the effect size and variance. how does a p-value in a small size of a pilot helps - p values are only meaningful following power calculations in this setting.

Response: We agree. In the revised manuscript, we have included focusing on point estimates, 95% confidence intervals and Cohen’s d effect sixes. These metrics will provide the required variance and magnitude of effect which will be used to calculate statistical power and sample size for the larger future trials. Updated in manuscript (Lines 389-393)

5) Also linked to 4, the analytical plan does not really match the cross-over design of the study. a mixed effects regression model can account for this reliably and is the preferred analytical approach. with 40 observations, however, it can fail to converge.

Response: Updated in manuscript (Lines 389-393)

**Minor**

1) Use of three statistical software packages is unusual and unexplained (minor but odd).

Response: Thank your for pointing that out. We have streamlined the protocol to state that all analyses will be conducted using SPSS to ensure consistency (Lines 392-393).

2) clarify if analyses will be ITT or per protocol.

Response: We have clarified in the “Statistical Analysis Plan” that the primary analysis will be conducted using an Intention-To-Treat (ITT) approach. (Lines 372-373)

3) There is an unusually large number of secondary and exploratory outcomes. some sort of prioritisation would help here.

Response: Conducting large-scale randomized controlled trials in healthcare settings can be resource-intensive and challenging; therefore, we focused on collecting comprehensive baseline and follow-up data within a single study. In addition, we included a brief diet quality screener (e.g., Mini-EAT) to explore whether such tools may be feasible for use as stand-alone measures in future studies. Several of the study outcomes will also be derived from a single dietary intake assessment, further supporting the inclusion of these measures. Importantly, the overall participant burden is expected to be minimal, as completion of all questionnaires together requires approximately 20 minutes.

**Reviewer 2:**

**Usual care is not well defined – what exactly do patients in the control group receive?**

Response: We thank the reviewer for this important point. We agree that "usual care" can vary between patients. At our center, there is currently no standardized dietary protocol or resource for IBD management; therefore, the control period is designed to reflect real-world clinical practice. To clarify this in the manuscript, we have mentioned it in the text (Lines: 237-243).

**Major Concerns**

The crossover design means control patients get the app after 30 days. This makes it hard to compare long-term effects between groups.

Response: We acknowledge that the crossover design limits the ability to make long-term between-group comparisons after the 30-day period. However, this design was intentionally selected to ensure that all participants eventually receive access to the intervention, which may help improve recruitment and participant retention. The initial monitoring period was intentionally kept short because the intervention is designed for regular, near-daily engagement with the app. We anticipated that consistent use over a shorter duration would be more meaningful and feasible than sporadic use over a prolonged study period. Accordingly, the primary comparative analysis will focus on the short-term effectiveness of the intervention, particularly the “Group × Time” interaction at the 30-day mark using a Linear Mixed Model.

In addition, our follow-up assessments are intended primarily to evaluate retention and sustained behavioral change rather than only immediate knowledge acquisition. Educational interventions may improve knowledge rapidly; however, our objective is to assess whether participants continue to apply what they have learned and whether any behavior changes are maintained over time. Therefore, the follow-up period was designed to evaluate the feasibility of educational retention, and the persistence of behavior change after initial exposure to the intervention.

No power calculation is provided. The authors say 40 is a "pilot" sample, but they should justify this number.

Response: As this is a pilot feasibility and usability study, a formal power calculation was not done. The sample size of n=40 (n=20 each arm) was chosen based on established recommendations for pilot studies, where it has been suggested that a minimum of 12 to 30 participants per arm are sufficient to estimate the variance required for a future power calculation (Hertzog, 2008; Julious, 2005). Updated in manuscript (Lines 173-176)

**Minor Concerns**

The title says "safety," but safety is not a primary outcome, and adverse event collection is not described in detail.

Response: We agree, safety has been taken out from the title and details on adverse event collection have been added (351-352)

Some references are outdated (e.g., nutrition prevalence data from 2003).

Response: Supporting more recent references have been added (Lines 67-68)

**BIBLIOGRAPHY:**

Hertzog, M. A. (2008). Considerations in determining sample size for pilot studies. *Research in Nursing & Health*, *31*(2), 180–191. https://doi.org/10.1002/NUR.20247

Julious, S. A. (2005). Sample size of 12 per group rule of thumb for a pilot study. *Pharmaceutical Statistics*, *4*(4), 287–291. https://doi.org/10.1002/PST.185;JOURNAL:JOURNAL:15391612;WGROUP:STRING:PUBLICATION

Lewis, J. D., Sandler, R. S., Brotherton, C., Brensinger, C., Li, H., Kappelman, M. D., Daniel, S. G., Bittinger, K., Albenberg, L., Valentine, J. F., Hanson, J. S., Suskind, D. L., Meyer, A., Compher, C. W., Bewtra, M., Saxena, A., Dobes, A., Cohen, B. L., Flynn, A. D., … Herfarth, H. (2021). A Randomized Trial Comparing the Specific Carbohydrate Diet to a Mediterranean Diet in Adults with Crohn’s Disease. *Gastroenterology*, *161*(3), 837. https://doi.org/10.1053/J.GASTRO.2021.05.047

Manski, S., Noverati, N., Policarpo, T., Rubin, E., & Shivashankar, R. (2024). Diet and Nutrition in Inflammatory Bowel Disease: A Review of the Literature. *Crohn’s & Colitis 360*, *6*(1). https://doi.org/10.1093/CROCOL/OTAD077
