# Supplementary material for: A pilot randomized trial on the usability and acceptability of an app (MyIBDDiet) to improve the self-management of anti-inflammatory diet for individuals with inflammatory bowel disease: A protocol paper
Source: PLoS One. 2026 Jul 2;21(7):e0353123. doi: 10.1371/journal.pone.0353123 (PMC13327250; doi:10.1371/journal.pone.0353123)
Supplement: Peer review history

# Peer review: Decision Letter 0

**DOI:** 10.1371/journal.pone.0353123.r001

**Authors:** Sophia Eugenia Martínez-Vázquez

19 May 2026

PONE-D-26-09025

A pilot randomized trial on the usability and safety of an app (MyIBDDiet) to improve the self-management of anti-inflammatory diet for individuals with inflammatory bowel disease: A protocol paper

PLOS One

Dear Dr. Wong,

Thank you for submitting your manuscript to PLOS ONE. After careful consideration, we feel that it has merit but does not fully meet PLOS ONE’s publication criteria as it currently stands. Therefore, we invite you to submit a revised version of the manuscript that addresses the points raised during the review process.

==============================

Dear authors,

Thank you for the opportunity to revise "A pilot randomized trial on the usability and safety of an app (MyIBDDiet) to improve the self-management of anti-inflammatory diet for individuals with inflammatory bowel disease: A protocol paper", here my comments:

1. It is necessary to stablish a period of time that reflects real changes proposed in the protocol; 30- 60 days could be few time, but please reference the period selected by you.

2. Clearly describe how you will measure behavioral changes, diet knowledge and safety of the app.

3. More references are needed to explain the eating behavior changes related to disease activity.

4. Suggest this studyas a exploratory in order to respond the primary objective an a randomized trial yo evaluate diet quality, disease control and quality of life.

5. Describe if other anthropometric measurements than height and weight will be recorded.

6. Please indicate why height need to be measured on each visit, do you expect any change?

7. Consider RM ANOVA to test changes over the time.

Thank you

==============================

Please submit your revised manuscript by june 2nd, 2026. If you will need more time than this to complete your revisions, please reply to this message or contact the journal office at plosone@plos.org. When you're ready to submit your revision, log on to https://www.editorialmanager.com/pone/ and select the 'Submissions Needing Revision' folder to locate your manuscript file.

Please include the following items when submitting your revised manuscript:

- A letter that responds to each point raised by the academic editor and reviewer(s). You should upload this letter as a separate file labeled 'Response to Reviewers'.
- A marked-up copy of your manuscript that highlights changes made to the original version. You should upload this as a separate file labeled 'Revised Manuscript with Track Changes'.
- An unmarked version of your revised paper without tracked changes. You should upload this as a separate file labeled 'Manuscript'.

If you would like to make changes to your financial disclosure, please include your updated statement in your cover letter. Guidelines for resubmitting your figure files are available below the reviewer comments at the end of this letter.

If applicable, we recommend that you deposit your laboratory protocols in protocols.io to enhance the reproducibility of your results. Protocols.io assigns your protocol its own identifier (DOI) so that it can be cited independently in the future. For instructions see: https://journals.plos.org/plosone/s/submission-guidelines#loc-laboratory-protocols. Additionally, PLOS ONE offers an option for publishing peer-reviewed Lab Protocol articles, which describe protocols hosted on protocols.io. Read more information on sharing protocols at https://plos.org/protocols?utm_medium=editorial-email&utm_source=authorletters&utm_campaign=protocols.

As the corresponding author, your ORCID iD is verified in the submission system and will appear in the published article. PLOS supports the use of ORCID, and we encourage all coauthors to register for an ORCID iD and use it as well. Please encourage your coauthors to verify their ORCID iD within the submission system before final acceptance, as unverified ORCID iDs will not appear in the published article. *Only*  the individual author can complete the verification step; PLOS staff *cannot*  verify ORCID iDs on behalf of authors.

We look forward to receiving your revised manuscript.

Kind regards,

Sophia Eugenia Martínez-Vázquez, Ph.D.

Academic Editor

PLOS One

Journal Requirements:

When submitting your revision, we need you to address these additional requirements.

1. Please ensure that your manuscript meets PLOS ONE's style requirements, including those for file naming. The PLOS ONE style templates can be found at

https://journals.plos.org/plosone/s/file?id=wjVg/PLOSOne_formatting_sample_main_body.pdf and

https://journals.plos.org/plosone/s/file?id=ba62/PLOSOne_formatting_sample_title_authors_affiliations.pdf

2. Thank you for stating the following financial disclosure:

“This study is funded with the Partnership for Research and Innovation in Health System (PRIHS 7) grant from Alberta Innovates.”

Please state what role the funders took in the study. If the funders had no role, please state: "The funders had no role in study design, data collection and analysis, decision to publish, or preparation of the manuscript."

If this statement is not correct you must amend it as needed.

Please include this amended Role of Funder statement in your cover letter; we will change the online submission form on your behalf.

3. Thank you for stating the following in the Acknowledgments Section of your manuscript:

“This study is funded with the Partnership for Research and Innovation in Health System (PRIHS 7) grant from Alberta Innovates. CMP is partially funded through Canada’s Research Chair Program.”

We note that you have provided funding information that is currently declared in your Funding Statement. However, funding information should not appear in the Acknowledgments section or other areas of your manuscript. We will only publish funding information present in the Funding Statement section of the online submission form.

Please remove any funding-related text from the manuscript and let us know how you would like to update your Funding Statement. Currently, your Funding Statement reads as follows:

“This study is funded with the Partnership for Research and Innovation in Health System (PRIHS 7) grant from Alberta Innovates.”

Please include your amended statements within your cover letter; we will change the online submission form on your behalf.

4. Thank you for stating the following in the Competing Interests section:

“I have read the journal's policy and the authors of this manuscript have the following competing interests: CMP declares speaking engagements and/or consultancy for Abbott Nutrition, Nestle Health Science, Nutricia, and Novo Nordisk.”

Please confirm that this does not alter your adherence to all PLOS ONE policies on sharing data and materials, by including the following statement: "This does not alter our adherence to PLOS ONE policies on sharing data and materials.” (as detailed online in our guide for authors http://journals.plos.org/plosone/s/competing-interests). If there are restrictions on sharing of data and/or materials, please state these. Please note that we cannot proceed with consideration of your article until this information has been declared.

Please include your updated Competing Interests statement in your cover letter; we will change the online submission form on your behalf.

5. We note that Figure 3 in your submission contain copyrighted images. All PLOS content is published under the Creative Commons Attribution License (CC BY 4.0), which means that the manuscript, images, and Supporting Information files will be freely available online, and any third party is permitted to access, download, copy, distribute, and use these materials in any way, even commercially, with proper attribution. For more information, see our copyright guidelines: http://journals.plos.org/plosone/s/licenses-and-copyright.

We require you to either (1) present written permission from the copyright holder to publish these figures specifically under the CC BY 4.0 license, or (2) remove the figures from your submission:

a. You may seek permission from the original copyright holder of Figure(s) [#] to publish the content specifically under the CC BY 4.0 license.

We recommend that you contact the original copyright holder with the Content Permission Form (http://journals.plos.org/plosone/s/file?id=7c09/content-permission-form.pdf) and the following text:

“I request permission for the open-access journal PLOS ONE to publish XXX under the Creative Commons Attribution License (CCAL) CC BY 4.0 (http://creativecommons.org/licenses/by/4.0/). Please be aware that this license allows unrestricted use and distribution, even commercially, by third parties. Please reply and provide explicit written permission to publish XXX under a CC BY license and complete the attached form.”

Please upload the completed Content Permission Form or other proof of granted permissions as an "Other" file with your submission.

In the figure caption of the copyrighted figure, please include the following text: “Reprinted from [ref] under a CC BY license, with permission from [name of publisher], original copyright [original copyright year].”

b. If you are unable to obtain permission from the original copyright holder to publish these figures under the CC BY 4.0 license or if the copyright holder’s requirements are incompatible with the CC BY 4.0 license, please either i) remove the figure or ii) supply a replacement figure that complies with the CC BY 4.0 license. Please check copyright information on all replacement figures and update the figure caption with source information. If applicable, please specify in the figure caption text when a figure is similar but not identical to the original image and is therefore for illustrative purposes only.

6. Please include captions for your Supporting Information files at the end of your manuscript, and update any in-text citations to match accordingly. Please see our Supporting Information guidelines for more information: http://journals.plos.org/plosone/s/supporting-information.

7. We note that the original protocol that you have uploaded as a Supporting Information file contains an institutional logo. As this logo is likely copyrighted, we ask that you please remove it from this file and upload an updated version upon resubmission.

8. If the reviewer comments include a recommendation to cite specific previously published works, please review and evaluate these publications to determine whether they are relevant and should be cited. There is no requirement to cite these works unless the editor has indicated otherwise.

[Note: HTML markup is below. Please do not edit.]

Reviewers' comments:

Reviewer's Responses to Questions

**Comments to the Author**

1. Does the manuscript provide a valid rationale for the proposed study, with clearly identified and justified research questions?

The research question outlined is expected to address a valid academic problem or topic and contribute to the base of knowledge in the field.

Reviewer #1: Yes

Reviewer #2: Yes

**********

2. Is the protocol technically sound and planned in a manner that will lead to a meaningful outcome and allow testing the stated hypotheses?

The manuscript should describe the methods in sufficient detail to prevent undisclosed flexibility in the experimental procedure or analysis pipeline, including sufficient outcome-neutral conditions (e.g. necessary controls, absence of floor or ceiling effects) to test the proposed hypotheses and a statistical power analysis where applicable. As there may be aspects of the methodology and analysis which can only be refined once the work is undertaken, authors should outline potential assumptions and explicitly describe what aspects of the proposed analyses, if any, are exploratory.

Reviewer #1: Yes

Reviewer #2: Yes

**********

3. Is the methodology feasible and described in sufficient detail to allow the work to be replicable?

Descriptions of methods and materials in the protocol should be reported in sufficient detail for another researcher to reproduce all experiments and analyses. The protocol should describe the appropriate controls, sample size calculations, and replication needed to ensure that the data are robust and reproducible.

Reviewer #1: Yes

Reviewer #2: Yes

**********

4. Have the authors described where all data underlying the findings will be made available when the study is complete?

The PLOS Data policy requires authors to make all data underlying the findings described in their manuscript fully available without restriction, with rare exception, at the time of publication. The data should be provided as part of the manuscript or its supporting information, or deposited to a public repository. For example, in addition to summary statistics, the data points behind means, medians and variance measures should be available. If there are restrictions on publicly sharing data—e.g. participant privacy or use of data from a third party—those must be specified.

Reviewer #1: Yes

Reviewer #2: Yes

**********

5. Is the manuscript presented in an intelligible fashion and written in standard English?

PLOS ONE does not copyedit accepted manuscripts, so the language in submitted articles must be clear, correct, and unambiguous. Any typographical or grammatical errors should be corrected at revision, so please note any specific errors here.

Reviewer #1: Yes

Reviewer #2: Yes

**********

6. Review Comments to the Author

Please use the space provided to explain your answers to the questions above and, if applicable, provide comments about issues authors must address before this protocol can be accepted for publication. You may also include additional comments for the author, including concerns about research or publication ethics.

You may also provide optional suggestions and comments to authors that they might find helpful in planning their study.

(Please upload your review as an attachment if it exceeds 20,000 characters)

Reviewer #1: As the statistical reviewer I will focus on methods and reporting

Major

1) the is no mention of the CONSORT statement.

2) in the methods section I expected a plan to deal with missing data. are any expected? if yes are multiple imputation approaches considered?

3) it was not clear to me what the multivariable analyses will add with a sample of this size. identifying predictors in a sample of 40, even as a secondary set of outcomes, is problematic. similarly about the PCA. if the authors need to keep those analyses, they need to explain they are strictly exploratory.

4) the main analysis, the comparison of the two group, is planned using a test that only reports statistical significance - how will that inform a future study? the authors need to obtain a measure of the effect size and variance. how does a p-value in a small size of a pilot helps - p values are only meaningful following power calculations in this setting.

5) Also linked to 4, the analytical plan does not really match the cross-over design of the study. a mixed effects regression model can account for this reliably and is the preferred analytical approach. with 40 observations, however, it can fail to converge.

Minor

1) Use of three statistical software packages is unusual and unexplained (minor but odd).

2) clarify if analyses will be ITT or per protocol.

3) There is an unusually large number of secondary and exploratory outcomes. some sort of prioritisation would help here.

Reviewer #2: This protocol describes a 60-day pilot trial testing a new diet app (MyIBDDiet) for people with IBD. The app helps users follow a Mediterranean diet and avoid processed foods. The study will enroll 40 patients, randomize them to app use or usual care, and measure usability as the main outcome.

Strengths

The app was co-designed with patients, which is very good

Mixed methods (questionnaires + interviews) will provide rich usability data

Clear rationale for focusing on Mediterranean diet based on recent guidelines

Includes objective markers like lab tests and stool samples, not just questionnaires

Weaknesses

The sample size is small (40 patients); authors correctly call this a pilot

No blinding—participants and researchers know who gets the app

Usual care is not well defined – what exactly do patients in the control group receive?

A six-month follow-up may be optimistic given that app use stops at 60 days

Major Concerns

The crossover design means control patients get the app after 30 days. This makes it hard to compare long-term effects between groups.

No power calculation is provided. The authors say 40 is a "pilot" sample, but they should justify this number.

Minor Concerns

The title says "safety," but safety is not a primary outcome, and adverse event collection is not described in detail.

Some references are outdated (e.g., nutrition prevalence data from 2003).

**********

7. PLOS authors have the option to publish the peer review history of their article (what does this mean?). If published, this will include your full peer review and any attached files.

If you choose “no”, your identity will remain anonymous but your review may still be made public.

**Do you want your identity to be public for this peer review?**  For information about this choice, including consent withdrawal, please see our Privacy Policy.

Reviewer #1: No

Reviewer #2: **Yes:**  Fatima Nadeem

**********

[NOTE: If reviewer comments were submitted as an attachment file, they will be attached to this email and accessible via the submission site. Please log into your account, locate the manuscript record, and check for the action link "View Attachments". If this link does not appear, there are no attachment files.]

To ensure your figures meet our technical requirements, please review our figure guidelines: https://journals.plos.org/plosone/s/figures

You may also use PLOS’s free figure tool, NAAS, to help you prepare publication quality figures: https://journals.plos.org/plosone/s/figures#loc-tools-for-figure-preparation.

NAAS will assess whether your figures meet our technical requirements by comparing each figure against our figure specifications.

# Author comment: Author response to Decision Letter 1

**DOI:** 10.1371/journal.pone.0353123.r002

11 Jun 2026

Academic Editor comments:

1. It is necessary to establish a period of time that reflects real changes proposed in the protocol; 30- 60 days could be few time, but please reference the period selected by you.

Response: We appreciate the reviewer’s feedback regarding the study duration. We selected the 60-day intervention period for several strategic reasons. Previous research on dietary interventions in IBD indicates that significant improvements in diet quality, inflammatory markers, and patient-reported outcomes can be typically observed within a 4- to 8-week window (Lewis et al., 2021; Manski et al., 2024). Additionally, as this is a pilot study, a 60-day timeframe is appropriate to assess core app engagement and "proof of concept" for behavioral change before committing to a longer-term definitive trial. Also, we want to ensure participants use the app on a regular, high-frequency basis. The 2-month duration was chosen to maximize consistent adherence, mitigating the risk of "engagement fatigue" often seen in longer digital health interventions. The protocol includes a 6-month follow-up period which we think help address sustained use or knowledge retention. This will allow us to evaluate long-term retention and the frequency of app use once the active trial phase has ended. The rationale has been updated in the manuscript as suggested (Lines 77-88)

2. Clearly describe how you will measure behavioral changes, diet knowledge and safety of the app.

Response: Thank you for this comment. It has been clarified as below:

Behavioral Changes: To capture a holistic view of behavior change and the resulting dietary shifts, we will use a mixed-methods approach. Objective changes will be assessed using the diet quality questionnaire (Mini-EAT) and changes in the nutrient intake data generated via ASA24 as well as the app backend data showing food tracking over time. Subjective changes will be captured through semi-structured interviews, allowing participants to describe the lived experience of altering food choices.

Dietary Knowledge: As there is currently no single validated questionnaire for testing specific dietary knowledge in this context (in particular for ultra-processed foods), we evaluate knowledge through patterns of change mainly the translation of knowledge into action. As having knowledge versus putting it to application are two different concepts and it is important for us to measure if the knowledge gained is being put to action. Therefore, we will measure it via changes in eating habits and also track changes in ultra-processed food intake.

Safety of using the app: There will be continuous monitoring and reporting of all adverse events (AEs) and serious adverse events (SAEs) throughout the study duration. Moreover, to ensure participant safety, individuals experiencing an active flare, having a bowel obstruction or a high risk of bowel obstruction related to dietary fiber changes have been excluded from the study protocol.

Updated in the manuscript as suggested (Lines 348-352)

3. More references are needed to explain the eating behavior changes related to disease activity.

Response: More references have been added to explain the eating behaviour changes related to disease activity (Lines 63-65)

4. Suggest this study as a exploratory in order to respond the primary objective and a randomized trial to evaluate diet quality, disease control and quality of life.

Response: We appreciate the reviewer’s perspective on the study's trajectory. We agree that a large-scale randomized controlled trial (RCT) is the gold standard for evaluating clinical outcomes such as diet quality, disease control, and quality of life. However, we believe the current study is a critical exploratory and foundational step. While the app was developed by academic researchers and patient partners who have conducted initial usability testing, it has not yet been deployed within a diverse, larger-scale population. Our primary objective at this stage is to integrate lived experience and optimize functionality. Following the successful completion of this exploratory phase and the refinement of the app based on participant feedback, we intend to conduct a future randomized trial with the primary aim to rigorously evaluate its effectiveness regarding clinical outcomes and quality of life.

5. Describe if other anthropometric measurements than height and weight will be recorded.

Response: We thank the reviewer for bringing this important point to light. We have decided to expand the anthropometrics methodology to include waist circumference and waist-to-hip ratio in the measurements, along with height and weight. However, I would like to bring to your attention that the primary goal of the app is not weight loss, but rather to focus on eating overall healthier foods. We have not anticipated using it for weight loss at this stage. In the future, as we expand the study, we may possibly include body composition parameters using ultrasonography or existing MRI and CT scans, but for now, we want to stay focused on the primary goal of healthy eating patterns.

Information updated in manuscript (Lines 280-281)

Please indicate why height need to be measured on each visit, do you expect any change?

Response: Thank you for your insightful comment. We agree height is not expected to change during the study in the population, we have revised the protocol to reflect the height will only be measured at baseline.

6. Consider RM ANOVA to test changes over the time.

Response: Thank you for the useful suggestion. We have updated our statistical plan based on feedback received from reviewer 1 and decided to use Linear Mixed-effects Model (LMM). As LMM can also handle missing data and accounts for within-subject correlation across multiple timepoints, we have decided to switch to this. Updated statistical analyses strategy in manuscript (Lines 370-437)

Reviewer 1:

Major

1) the is no mention of the CONSORT statement.

Response: Thank you for this observation. We have followed the SPIRIT (Standard Protocol Items: Recommendations for Interventional Trials) statement which is internationally recognized guideline specifically designed for the reporting of study protocols. Since we are only reporting the study design and the protocol in this manuscript, we decided to report the SPIRIT checklist and will be reporting the CONSORT checklist once we publish the results and outcomes.

2) in the methods section I expected a plan to deal with missing data. are any expected? if yes are multiple imputation approaches considered?

Response: Thank you for your feedback. We have clarified the plan for missing data in the revised manuscript. We will utilize the restricted maximum likelihood approach of Linear Mixed Models (LMM) to handle the missing data. However, if missing data exceeds 20%, we will implement Multiple Imputation by Chained Equations (MICE) to ensure robustness. Updated in manuscript (Lines 378-388)

3) it was not clear to me what the multivariable analyses will add with a sample of this size. identifying predictors in a sample of 40, even as a secondary set of outcomes, is problematic. similarly, about the PCA. if the authors need to keep those analyses, they need to explain they are strictly exploratory.

Response: This is an excellent point. We have revised the manuscript to clearly state that the multivariable predictors and PCA will be strictly exploratory. The purpose of these analyses will be to generate hypothesis to be tested in future larger trials. Updated in manuscript (Lines 436-437)

4) the main analysis, the comparison of the two group, is planned using a test that only reports statistical significance - how will that inform a future study? the authors need to obtain a measure of the effect size and variance. how does a p-value in a small size of a pilot helps - p values are only meaningful following power calculations in this setting.

Response: We agree. In the revised manuscript, we have included focusing on point estimates, 95% confidence intervals and Cohen’s d effect sixes. These metrics will provide the required variance and magnitude of effect which will be used to calculate statistical power and sample size for the larger future trials. Updated in manuscript (Lines 389-393)

5) Also linked to 4, the analytical plan does not really match the cross-over design of the study. a mixed effects regression model can account for this reliably and is the preferred analytical approach. with 40 observations, however, it can fail to converge.

Response: Updated in manuscript (Lines 389-393)

Minor

1) Use of three statistical software packages is unusual and unexplained (minor but odd).

Response: Thank your for pointing that out. We have streamlined the protocol to state that all analyses will be conducted using SPSS to ensure consistency (Lines 392-393).

2) clarify if analyses will be ITT or per protocol.

Response: We have clarified in the “Statistical Analysis Plan” that the primary analysis will be conducted using an Intention-To-Treat (ITT) approach. (Lines 372-373)

3) There is an unusually large number of secondary and exploratory outcomes. some sort of prioritisation would help here.

Response: Conducting large-scale randomized controlled trials in healthcare settings can be resource-intensive and challenging; therefore, we focused on collecting comprehensive baseline and follow-up data within a single study. In addition, we included a brief diet quality screener (e.g., Mini-EAT) to explore whether such tools may be feasible for use as stand-alone measures in future studies. Several of the study outcomes will also be derived from a single dietary intake assessment, further supporting the inclusion of these measures. Importantly, the overall participant burden is expected to be minimal, as completion of all questionnaires together requires approximately 20 minutes.

Reviewer 2:

Usual care is not well defined – what exactly do patients in the control group receive?

Response: We thank the reviewer for this important point. We agree that "usual care" can vary between patients. At our center, there is currently no standardized dietary protocol or resource for IBD management; therefore, the control period is designed to reflect real-world clinical practice. To clarify this in the manuscript, we have mentioned it in the text (Lines: 237-243).

Major Concerns

The crossover design means control patients get the app after 30 days. This makes it hard to compare long-term effects between groups.

Response: We acknowledge that the crossover design limits the ability to make long-term between-group comparisons after the 30-day period. However, this design was intentionally selected to ensure that all participants eventually receive access to the intervention, which may help improve recruitment and participant retention. The initial monitoring period was intentionally kept short because the intervention is designed for regular, near-daily engagement with the app. We anticipated that consistent use over a shorter duration would be more meaningful and feasible than sporadic use over a prolonged study period. Accordingly, the primary comparative analysis will focus on the short-term effectiveness of the intervention, particularly the “Group × Time” interaction at the 30-day mark using a Linear Mixed Model.

In addition, our follow-up assessments are intended primarily to evaluate retention and sustained behavioral change rather than only immediate knowledge acquisition. Educational interventions may improve knowledge rapidly; however, our objective is to assess whether participants continue to apply what they have learned and whether any behavior changes are maintained over time. Therefore, the follow-up period was designed to evaluate the feasibility of educational retention, and the persistence of behavior change after initial exposure to the intervention.

No power calculation is provided. The authors say 40 is a "pilot" sample, but they should justify this number.

Response: As this is a pilot feasibility and usability study, a formal power calculation was not done. The sample size of n=40 (n=20 each arm) was chosen based on established recommendations for pilot studies, where it has been suggested that a minimum of 12 to 30 participants per arm are sufficient to estimate the variance required for a future power calculation (Hertzog, 2008; Julious, 2005). Updated in manuscript (Lines 173-176)

Minor Concerns

The title says "safety," but safety is not a primary outcome, and adverse event collection is not described in detail.

Response: We agree, safety has been taken out from the title and details on adverse event collection have been added (351-352)

Some references are outdated (e.g., nutrition prevalence data from 2003).

Response: Supporting more recent references have been added (Lines 67-68)

BIBLIOGRAPHY:

Hertzog, M. A. (2008). Considerations in determining sample size for pilot studies. Research in Nursing & Health, 31(2), 180–191. https://doi.org/10.1002/NUR.20247

Julious, S. A. (2005). Sample size of 12 per group rule of thumb for a pilot study. Pharmaceutical Statistics, 4(4), 287–291. https://doi.org/10.1002/PST.185;JOURNAL:JOURNAL:15391612;WGROUP:STRING:PUBLICATION

Lewis, J. D., Sandler, R. S., Brotherton, C., Brensinger, C., Li, H., Kappelman, M. D., Daniel, S. G., Bittinger, K., Albenberg, L., Valentine, J. F., Hanson, J. S., Suskind, D. L., Meyer, A., Compher, C. W., Bewtra, M., Saxena, A., Dobes, A., Cohen, B. L., Flynn, A. D., … Herfarth, H. (2021). A Randomized Trial Comparing the Specific Carbohydrate Diet to a Mediterranean Diet in Adults with Crohn’s Disease. Gastroenterology, 161(3), 837. https://doi.org/10.1053/J.GASTRO.2021.05.047

Manski, S., Noverati, N., Policarpo, T., Rubin, E., & Shivashankar, R. (2024). Diet and Nutrition in Inflammatory Bowel Disease: A Review of the Literature. Crohn’s & Colitis 360, 6(1). https://doi.org/10.1093/CROCOL/OTAD077

Attachment Submitted filename: Response to Reviewers_MyIBDDiet Protocol.docx

# Peer review: Decision Letter 1

**DOI:** 10.1371/journal.pone.0353123.r003

**Authors:** Sophia Eugenia Martínez-Vázquez

18 Jun 2026

A pilot randomized trial on the usability and acceptability of an app (MyIBDDiet) to improve the self-management of anti-inflammatory diet for individuals with inflammatory bowel disease: A protocol paper

PONE-D-26-09025R1

Dear Dr. Wong,

We’re pleased to inform you that your manuscript has been judged scientifically suitable for publication and will be formally accepted for publication once it meets all outstanding technical requirements.

Within one week, you’ll receive an e-mail detailing the required amendments. When these have been addressed, you’ll receive a formal acceptance letter and your manuscript will be scheduled for publication.

An invoice will be generated when your article is formally accepted. Please note, if your institution has a publishing partnership with PLOS and your article meets the relevant criteria, all or part of your publication costs will be covered. Please make sure your user information is up-to-date by logging into Editorial Manager at Editorial Manager® and clicking the ‘Update My Information' link at the top of the page. For questions related to billing, please contact billing support.

If your institution or institutions have a press office, please notify them about your upcoming paper to help maximize its impact. If they’ll be preparing press materials, please inform our press team as soon as possible -- no later than 48 hours after receiving the formal acceptance. Your manuscript will remain under strict press embargo until 2 pm Eastern Time on the date of publication. For more information, please contact onepress@plos.org.

Kind regards,

Sophia Eugenia Martínez-Vázquez, Ph.D.

Academic Editor

PLOS One

Additional Editor Comments (optional):

Dear Authors,

Thank you for your detailed responses and overall the improvements in your document, It is now a robust protocol. Congratulations.

Reviewers' comments:

Reviewer's Responses to Questions

**Comments to the Author**

1. Does the manuscript provide a valid rationale for the proposed study, with clearly identified and justified research questions?

The research question outlined is expected to address a valid academic problem or topic and contribute to the base of knowledge in the field.

Reviewer #1: Yes

**********

2. Is the protocol technically sound and planned in a manner that will lead to a meaningful outcome and allow testing the stated hypotheses?

The manuscript should describe the methods in sufficient detail to prevent undisclosed flexibility in the experimental procedure or analysis pipeline, including sufficient outcome-neutral conditions (e.g. necessary controls, absence of floor or ceiling effects) to test the proposed hypotheses and a statistical power analysis where applicable. As there may be aspects of the methodology and analysis which can only be refined once the work is undertaken, authors should outline potential assumptions and explicitly describe what aspects of the proposed analyses, if any, are exploratory.

Reviewer #1: Yes

**********

3. Is the methodology feasible and described in sufficient detail to allow the work to be replicable?

Descriptions of methods and materials in the protocol should be reported in sufficient detail for another researcher to reproduce all experiments and analyses. The protocol should describe the appropriate controls, sample size calculations, and replication needed to ensure that the data are robust and reproducible.

Reviewer #1: Yes

**********

4. Have the authors described where all data underlying the findings will be made available when the study is complete?

The PLOS Data policy requires authors to make all data underlying the findings described in their manuscript fully available without restriction, with rare exception, at the time of publication. The data should be provided as part of the manuscript or its supporting information, or deposited to a public repository. For example, in addition to summary statistics, the data points behind means, medians and variance measures should be available. If there are restrictions on publicly sharing data—e.g. participant privacy or use of data from a third party—those must be specified.

Reviewer #1: Yes

**********

5. Is the manuscript presented in an intelligible fashion and written in standard English?

PLOS ONE does not copyedit accepted manuscripts, so the language in submitted articles must be clear, correct, and unambiguous. Any typographical or grammatical errors should be corrected at revision, so please note any specific errors here.

Reviewer #1: Yes

**********

6. Review Comments to the Author

Please use the space provided to explain your answers to the questions above and, if applicable, provide comments about issues authors must address before this protocol can be accepted for publication. You may also include additional comments for the author, including concerns about research or publication ethics.

You may also provide optional suggestions and comments to authors that they might find helpful in planning their study.

(Please upload your review as an attachment if it exceeds 20,000 characters)

Reviewer #1: All comments have been addressed and the authors have made meaningful changes to the paper in response to them.

**********

7. PLOS authors have the option to publish the peer review history of their article (what does this mean?). If published, this will include your full peer review and any attached files.

If you choose “no”, your identity will remain anonymous but your review may still be made public.

**Do you want your identity to be public for this peer review?**  For information about this choice, including consent withdrawal, please see our Privacy Policy.

Reviewer #1: No

**********

# Editor report: Acceptance letter

**DOI:** 10.1371/journal.pone.0353123.r004

**Authors:** Sophia Eugenia Martínez-Vázquez

PONE-D-26-09025R1

PLOS One

Dear Dr. Wong,

I'm pleased to inform you that your manuscript has been deemed suitable for publication in PLOS One. Congratulations! Your manuscript is now being handed over to our production team.

At this stage, our production department will prepare your paper for publication. This includes ensuring the following:

* All references, tables, and figures are properly cited

* All relevant supporting information is included in the manuscript submission,

* There are no issues that prevent the paper from being properly typeset

You will receive further instructions from the production team, including instructions on how to review your proof when it is ready. Please keep in mind that we are working through a large volume of accepted articles, so please give us a few days to review your paper and let you know the next and final steps.

Lastly, if your institution or institutions have a press office, please let them know about your upcoming paper now to help maximize its impact. If they'll be preparing press materials, please inform our press team within the next 48 hours. Your manuscript will remain under strict press embargo until 2 pm Eastern Time on the date of publication. For more information, please contact onepress@plos.org.

You will receive an invoice from PLOS for your publication fee after your manuscript has reached the completed accept phase. If you receive an email requesting payment before acceptance or for any other service, this may be a phishing scheme. Learn how to identify phishing emails and protect your accounts at https://explore.plos.org/phishing.

If we can help with anything else, please email us at customercare@plos.org.

Thank you for submitting your work to PLOS ONE and supporting open access.

Kind regards,

PLOS ONE Editorial Office Staff

on behalf of

Dr. Sophia Eugenia Martínez-Vázquez

Academic Editor

PLOS One
